# Costs over benefits: mind wandering in sporting performance

**DOI:** 10.3389/fpsyg.2024.1347561

**Published:** 2024-07-01

**Authors:** Jieling Li, Yafang Liu, Shuangpeng Xue, Bao Tian

**Affiliations:** ^1^School of Physical Education, Hebei Normal University, Shijiazhuang, China; ^2^Key Laboratory of Measurement and Evaluation in Exercise Bioinformation of Hebei Province, Shijiazhuang, China; ^3^Physical Education Postdoctoral Research Station, Hebei Normal University, Shijiazhuang, China; ^4^Department of Physical Education, Tangshan Normal University, Tangshan, China; ^5^School of Psychology, Capital Normal University, Beijing, China

**Keywords:** athlete, mind wandering, grounded theory, systems thinking, performance

## Abstract

**Introduction:**

Athletes’ mind wandering during competition has positive and negative effects. The purpose of this study was to explore the reason for these bidirectional effects.

**Methods:**

We recruited 51 athletes from China to take part in semi-structured interviews in which we explored their experiences of mind wandering in competition. We used grounded theory combined with systems thinking to complete the data analysis and theoretical construction.

**Results:**

Results showed that the influence of mind wandering on sporting performance was dynamically influenced by “mind wandering source,” “competition anxiety,” “content of mind wandering,” “attentional resources” and “attentional control,” resulting in our development of the theory of “mind wandering in sporting performance (MWSP).” The above factors determine how mind wandering occurs and how it affects the competition.

**Discussion:**

Although the occurrence of mind wandering during competition has positive effects, its negative effects cannot be ignored, which may lead to athletes losing the race (costs over benefits). Potential strategies focusing on the mitigation of negative effects and promotion of positive effects of mind wandering are discussed.

## Introduction

An athlete’s mind often wanders in competition, negatively affecting the athlete. In the 2020 Tokyo Olympics, Sergey Kulish, an athlete representing Ukraine, was the first to be eliminated from the men’s 50 m rifle three-position final because he mistakenly shot the opponent’s target. After the match, Kulish felt very frustrated; he said he felt uncomfortable in his clothes, causing his mind to wander (Guang Ming Net, 2022).^1^ In the 2022 Beijing Winter Olympics, Chinese short-track speed skater Ren Ziwei was eventually disqualified due to a foul in the semi-final. Ren said that he had been thinking about the final and did not pay attention to the details of the race (China News, 2008).^2^

Psychological studies refer to this phenomenon as mind wandering (MW). MW is a familiar everyday experience (Killingsworth and Gilbert, 2010; Sherwood, 2014; Seli et al., 2018a), so considerable research efforts have been made to understand its effects (Blondé et al., 2022; Deng et al., 2022; Soemer et al., 2023; Yoshida et al., 2023). Considerable controversy surrounds the concept of MW (Christoff et al., 2018; Seli et al., 2018b). Seli et al. (2018b) argued that MW encompasses a broad range of phenomena, it is an umbrella term like “cognition” and “creativity,” and it is best considered from a family-resemblances perspective. Therefore, the researcher should always define the scope of MW before conducting a study. MW is a situation in which executive control shifts away from a primary task to the processing of personal goals, individuals lack control in this process (Smallwood and Schooler, 2006). The contents of MW arise from intrinsic changes that occur within individuals (Smallwood and Schooler, 2015). MW is different from self-talk and self-regulatory for its characteristic that lack of control. In the present study, we consider the term MW to refer to a mind that is not tied to the sporting tasks that athletes perform in competition but rather becomes focused on an internal thought.

The study of MW in the sports context is in its infancy. But researchers have found that not all MW is harmful. Regarding positive impacts, Miś and Kowalczyk (2020) found that participants’ moods improved after long-run training, and the positive emotional shift became more pronounced when their minds wandered toward the future. In addition, it is found that MW was related to helpful distraction, beneficial emotions and sudden insight, as well as to detrimental distracion and debilitative emotions by investigating its specific effects in sports (Latinjak, 2018). Thus, the influence of MW in the sports context is bi-directional. The context regulation hypothesis is a theory developed to analyze the effects of MW and suggests that explaining the effects of MW requires a focus on the task context (Smallwood and Andrews-Hanna, 2013). Therefore, the present study will answer the question in the competition context.

The purpose of grounded theory is to build a theory based on data, emphasizing deeper investigation into the reasons behind the behavior (Glaser, 1978). Grounded theory is suitable for fields that lack explanation and have not been researched and theorized (Flick, 2021), and it is especially suitable for solving the problems of this study. Grounded theory is a systematic and flexible approach to collecting and analyzing qualitative data to construct theories that are grounded in the data (Charmaz, 2006; Corbin and Strauss, 2015). Therefore, we conducted this study based on grounded theory to explain MW’s bidirectional effects and develop a theory that may provide scientific guidance for intervention in athletes’ MW.

## Materials and methods

### Participants

The selection of participants was based on the following considerations: First, only athletes themselves know the phenomenon of MW in competition best; other stakeholders such as coaches cannot accurately observe and measure it, let alone feel the process of its occurrence and influence. Therefore, only athletes were selected as the participants. The introspective method requires participants to report their mental activity and then draw a certain psychological conclusion by analyzing the reported data. Introspective method was chosen to guarantee that MW is indeed being investigated, and to assess both its occurrence and influence. Second, the total number of participants was determined by the principle of theoretical saturation, that is, the point at which new participants could neither provide new properties of a category nor generate new insights about the theory (Bryant and Charmaz, 2007). Third, different sporting events must be considered. Cases with high information intensity were selected on the basis of the principle of intensity sampling. The sporting events that are prone to MW must first be identified. Shooting is a type of static sport that requires a high ability of continuous attention. Shooters need to process less information on the field, and their tasks have lower cognitive loads. According to Cheyne et al. (2009), when the task difficulty is low (the cognitive load is low), more cognitive resources will be used for MW. Therefore, in the present study, shooters were first selected for data collection, and then athletes in other sports were selected for comparison and validation. Specifically, sampling was adopted in three steps. In the first step, 14 shooters were selected. Then, to enrich and compare the research results of the first step, considering the differences in gender and sports level, we selected 19 more shooters in the second step. In the third step, aiming to ensure the universality of the results, we selected 18 athletes from 9 other sporting events, namely, golf (1 person), archery (1 person), gymnastics (1 person), dance sports (1 person), table tennis (2 people), basketball (2 people), volleyball (2 people), tennis (3 people), and track and field (5 people). The participants were from professional sport teams and competitive sport schools in six cities in China, namely, Beijing, Tianjin, Shijiazhuang, Chongqing, Zhengzhou, and Wenzhou. They must train for more than 10 h a week and had to participate in competitions at or above the provincial level. A total of 51 athletes were selected for the study, comprising 26 male and 25 female athletes, aged 15–35 years old. Of the athletes, 2 were at the national elite level, 18 at national level 1, 22 at national level 2, and 9 athletes below national level 2. Approval for the research was obtained from the relevant research ethics committee (2023LLSC031).

### Data collection

Semi-structured interviews were applied to collect data. First, we introduced the research purpose and the meaning of MW to the athletes, and we explained that the findings would be confidential. The interviews were recorded after obtaining the participant’s consent. The interview duration varied from 11 to 90 min, average time was about 27 min. The interviews mainly included the context of MW, contents of MW, and MW coping methods and effects. Athletes needed to recall MW experiences from previous competitions. Interview guidelines involving the entire process of MW occurring, specific questions included “Under what circumstances will your mind wander during a competition?”, “What are you thinking when your mind is wandering?”, “What do you do when your mind wanders during the competition?”, and “What is the impact of MW on you?”. After the interview, audio-taped data were converted into verbatim transcripts in Chinese. Then, a corresponding number was set for each interviewed athlete (for example, 01 represents the first athlete interviewed). Then, NVivo 11.0 qualitative analysis software was used to encode the text data.

### Data analysis

Grounded theory advocates the development of theories based on the collected data in an inductive way (Glaser, 1998). Grounded theory has been developed over the years and has been recognized by academics, and different variants of thought have been formed. It is not a rigid methodology that is in lockstep, as are some statistical methodologies (Glaser, 2008). As long as the grounded advocacy is maintained, specific research methods and techniques can be flexibly adapted, innovated, and improved according to different research areas, problems, and contexts, and they can be used more widely and effectively to bring their true vitality to practice (Glaser, 1998; Flick, 2021).

Systems thinking can be applied to grounded theory. Systems thinking is the analysis of the interplay of factors from a systems perspective. The value of systems thinking is that it recognizes that factors do not exist in isolation, and that related factors interact with each other and are ultimately presented in a systematic way (Sherwood, 2014). The relationships among variables presented by systems thinking are dynamic and can change the static description previously presented by grounded theory.

**FIGURE 1:**
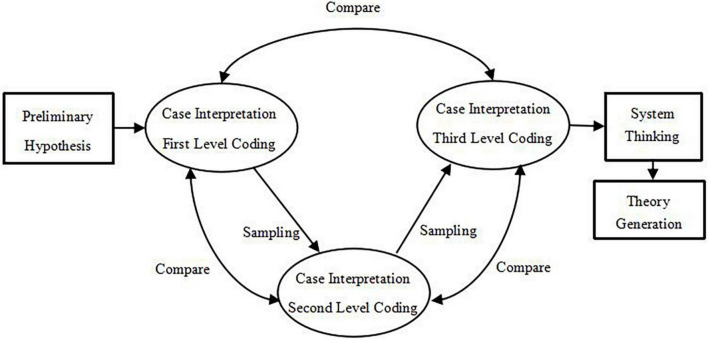
The research process of combining grounded theory with system thinking.

In this study, we followed the spirit of grounded theory and used three-level coding combined with systems thinking to complete the theoretical construction. The research process is shown in Figure 1.

We mainly used the grounded theory introduced by Strauss and Corbin (1990) as a realist–interpretivist philosophical perspective (Weed, 2016). But the three-level coding is simplified to three processes after local modification (Chen and Wang, 2021), forming categories, determining the core category, and determining the associated categories. At the same time, a constant comparison method was used, alternating among data collection, data analysis, and theory generation.

The primary function of the first level of coding is divided into three steps. The first step is labeling, or conceptual naming of the data. The second step is combining similar or identical codes into categories. The third step is identifying the attributes and dimensions of the categories. This approach focuses on the description of the nature of the category, highlighting the purpose of the explanation of the phenomenon by grounded theory. At the first level we obtained results as shown in Table 1. Using “MW Source” for example, the presence of spectators mentioned by the athletes will be MW, which belongs to the attribute of “interference from others” in the category of “MW Source,” and the dimension of its change is from strong to weak.

**TABLE 1:** Categories, attributes, and dimensions.

Category	Attributes	Dimension
MW source	Interference from others (18.10)	Strong, weak
	Time constraint (25.86)	Urgent, non-urgent
	Performance fluctuation (49.14)	Good, bad
	Environmental acceptance (6.90)	Acceptable, unacceptable
Competition anxiety	Somatic anxiety (33.90)	High, low
	Cognitive anxiety (66.10)	High, low
Content of MW	Relationship to the competition (25.52)	Relevant, irrelevant
	Time orientation (22.89)	Past, present, future
	Emotional valence (5.25)	Positive, negative
	Intention (15.76)	Yes, no
	Meta-consciousness (24.95)	Yes, no
	Degree (2.06)	Deep, shallow
	Duration (3.56)	Long, short
Attentional resource	Required for MW (48.28)	More, less
	Required for sport (51.72)	More, less
Attentional control	Attentional shifting (54.55)	Strong, weak
	Attentional focusing (45.45)	Strong, weak
Costs over benefits	Positive effect (21.21)	Strong, weak
	Negative effect (78.79)	Strong, weak

Numbers in parentheses indicate percentage of the number of codes for its category. MW, mind wandering.

For the second level of coding, we referred to Glaser’s approach of directly finding the core category (Glaser, 1978). In addition, we used the technique of a clarifying storyline to summarize the research. We found the core category of this study was “costs over benefits.” The storyline is expressed in level 2 coding of the results section.

For the third level of coding, we referred to advice from Strauss and Corbin (1990) that associated all the important categories with each other, applying systems thinking to do so. This method makes it easier for us to discuss the interrelationship between variables, which are categories in this study. The basic approach of systems thinking (Sherwood, 2014) is as follows: the interrelationship between variables is represented by connections, which are of two types: S- and O-type. In S-type, the growth of one variable leads to the growth of another variable. In O-type, the growth of one variable leads to the decline of another variable. The connections do not exist in isolation; they may contain a loop, that is, a feedback loop. Each variable in the loop is both a cause and a result and is influenced by and influences other variables. A feedback loop is also divided into two categories: reinforcing and balancing feedback. Reinforcing feedback accelerates the process of change. Benign and malignant cycles are forms of change, so the direction of change is consistent among variables and is known as R-loop. Balancing feedback is the process of regulation, in the form of fluctuations around the desired levels up and down. The direction of change is opposite among variables and is known as B-loop. Therefore, the number of O-type connections in the loop determines the type of loop: an even number of O-types is an R-loop, and an odd number of O-types is a B-loop. The interrelationship between categories is expressed in level 3 coding of the results section.

### Rigor

The rigor of this research is reflected in the entire study process of sampling, data collection, data analysis, and theory generation.

In the sampling process, the principles of intensity sampling, and theoretical saturation were followed. The analysis of research data alternates with sampling to avoid problems in research quality caused by one-time sampling. We trained interviewers to ensure consistency in data collection, and the training emphasized interview skills and content.

A total of two researchers participated in the data analysis, which was done independently and without interference from others. We also kept an open attitude during the analysis of the data, and the results were grounded in the original data. To improve the reliability of the data analysis, this study compared the coded results among athletes of different sports. Memos were used throughout the research process to record at any time the generated ideas, codes, and associations. The purpose of the memos is to actively think about the original data, stimulate inspiration, and generate new concepts and relationships to facilitate theory generation. These memos also helped us find loopholes in data collection and analysis and ensured that the data were examined from different perspectives, enhancing the validity of the entire study.

Member checking and non-participant checking were applied to test the study results. The codes and results were returned to the participants, and they were asked to judge whether the results accurately reflected their experiences. In this study, four athletes were selected to provide feedback on the results. For example, the dimensions of “physical environment acceptability” were originally expressed as “strong” and “weak,” but later the athletes determined that the appropriate expressions were “acceptable” and “unacceptable.” The results of the coding of the athletes’ MW were initially coded as “cognitive resources,” but we ultimately changed them to “attentional resources” after athlete insights. We also provided feedback to non-participants, including sports psychology majors (five people) and organizational behavior majors (two people), to control and correct coders’ subjective biases and to verify the authenticity of the coding results and the rationality of theory generation. For example: the naming of “costs over benefits” and “MW source” was decided through discussion with and verification by sports psychology colleagues. The theory of athletes’ MW was determined by reflection and judgment, and specific explanations were made in terms of the symbols and meanings of the loops. Finally, they were verified from the aspects of logic and comprehensibility.

## Results

### Level 1 coding: determining the categories, attributes, and dimensions

The primary function of the first level of coding is the formation of categories, as shown in Table 1.

#### MW source

In competition, there are four main MW sources: (1) Interference from others. Leaders, spectators, and opponents can cause MW in the athlete. As illustrated by 26: “*When leaders are around (resulting in MW), maybe this causes pressure in my mind.*” As 05 described: “*When you care about the spectators behind you and what they’re thinking.*” and “*Sometimes, when I meet opponents in front of or behind me, MW will occur.*” (2) Time constraint. The competition period results in urgent and non-urgent feelings in athletes, both of which tend to cause MW. Some events require athletes to complete a task within a short time, and the sense of time urgency can lead to MW in athletes. As conveyed by 23: “*Every shot has a time limit, and you have to make sure that you shoot within that time. So I would think… (MW).*” However, sometimes idle situations occur during the competition when the sense of urgency of the athlete decreases or disappears. This was expressed by 40: “*Four people stand like this when they’ve been pulling the oblique line. I have nothing to do with the ball, and I’ve been waiting for my partners in the completion of this task. If the time is a little longer, under these circumstances, MW occurs.*” (3) Performance fluctuation. When sporting performance does not match an athlete’s expectations (good or bad), MW easily arises. As 20 illustrated, “*When I played, I did not play well, and I hit several unsatisfactory ring numbers, easily resulting in MW.*” Respondent 30 also emphasized, “*If the result is better, I might think more.*” (4) Environmental acceptance. MW can be easily caused by the sound, temperature, and light on the field being beyond the acceptable range for the athlete. For example, 26 said, “*I may also wander when the audience makes heckling sounds.*” Respondent 21 also emphasized, “*It’s easy to wander if it’s quiet.*” As 08 explained: “*For example, if it’s hot or cold, you can’t accept the environment and you’ll be MW.*” Notably, the “audience” in the first MW source “interference from others” is different from the audience in environmental acceptance because the former emphasizes that the athlete cares about the audience’s evaluation of them, while the latter emphasizes the athletes’ receptivity to the sound produced by the audience.

#### Competition anxiety

When the level of competition anxiety is high, the athlete’s mind tends to wander. As stated by 32: “*When I am competing, I am especially anxious, and I will think about the results or the outcome, and I can’t concentrate on the task (MW).*” Respondent 05 said, “*When I shoot at points, I suddenly become a different person, that is, my body starts to shake and I can’t aim at the target at all. Then, when I am nervous, I would bare my teeth and make a sound, and I would worry about what to do, ‘What should I do here?’ (MW).*” In these cases, nervousness and worry represent cognitive anxiety, while shaking and making noises represent somatic anxiety. MW can also lead to an increase in athletes’ competition anxiety. Respondent 07 stated: “*A wandering mind easily upsets the mood.*” and 05 said: “*Wandering also increases my tension even more.*” A mutual influence is observed between the athletes’ MW and competition anxiety.

#### Content of MW

First, an athlete’s content of MW can exhibit a crossover of certain attributes. For example, 38 recalled: “*I look at this pedal (*track and field*) and I think about (MW) what if I don’t step on it, and I panic.*” Typical features are the “relevant” dimension in the “relationship to the competition” attribute, the “future” dimension in the “time orientation” attribute, the “negative” dimension in the “emotional valence” attribute, and the “yes” dimension in the “meta-consciousness” attribute. Respondent 11 stated: “*If I calculate the scores and think about the outcome of the competition, I can’t get out of [MW] because I have gotten myself into it and my head is not clear.*” The expressions are the “relevant” dimension in the “relationship to the competition” attribute, the “yes” dimension in the “meta-consciousness” attribute, “deep” in the “degree” attribute, and “long” in the duration attribute. Respondent 35 said: “*I avoid being so nervous. I just think about other things, like what we’re going to eat if we win and what’s going to happen.*” The expressions are the “irrelevant” dimension of the “relationship to the competition” attribute, the “future” dimension of the “time orientation” attribute, the “yes” dimension of the “intention” attribute, and the “positive” dimension in the “emotional valence” attribute.

Different content of MW affects athletes in different ways, but positive and negative effects co-exist. For example, 13 said, “*A good impact on you is when you think more positively. But when you think about not playing well or some other miscellaneous things, it will have some negative impacts on you.*” Respondent 46 explained: “*It’s not good for you if you’re thinking about life and not focusing on the ball. It’s not good for your performance in the game.*” But 17 also said, “*Thinking about something else will cause me to relax, and it’s not so tense.*”

#### Attentional resources

Mind wandering in competition often occurs when the demand for attentional resources for task is low so that some attentional resources can be allocated to MW. For example, 40 said: “*In singles matches, instances of MW are still quite few. Singles matches are one-on-one, in which the ball goes over and immediately comes back.*” Respondent 28 stated: “*[MW] should be in the preparation session.*” Respondent 22 explained: “*It’s easier to get MW with slow shots because it’s so slow.*” As regards when MW occurs, 19 said: “*Probably during practice before the match.*” As 11 described: “*I feel like you shouldn’t be able to do that much at the same time because human ‘energy’ is limited. You’re just going to have to keep an eye on the flat square, and you don’t have the ‘energy’ to think about how your index finger is going to pull the trigger.*” Respondent 49 also explained: “*Attention is also limited and may require some ‘energy’ as a base.*” In response to this question, we did post-interview member checks. After explaining the concept of attentional resources to the athletes, we finally recognized that the “energy” they were talking about was attentional resources. Athletes’ MW will therefore occupy attention resources. Individual attention resources are limited; both sport tasks and MW occupy attention resources. Thus, the phenomenon described by these athletes will occur: when the demand for attention resources for task is low, MW easily occurs.

#### Attentional control

In a competition, athletes use the method of attentional control to prevent MW when they encounter MW sources. For example, 07 stated: “*I just let myself not think about [MW] and just focus on the shot.*” Respondent 23 said: “*I try to isolate everything as much as I can. I just don’t let [the MW sources] go into my head.*” Respondent 08 recalled: “*We had electronic targets by that time and there would be a display in front of it. I would take a piece of paper and cover up that ring count to keep myself from looking at it.*” Athletes also have their own specific ways to control attention. For example, 40 said, “*It’s about paying attention to whether you’re close to wandering, letting yourself relax, and then pulling back.*” Respondent 04 described: “*[I give myself] just a little pinch, just a small action. I feel the pain, then I will be reminded that I am in the game, and my state will be a little better.*” Respondent 04 shared: “*I will stop, put down the gun, then sit on the bench to rest for a while, causing my brain to think again. I then go through my movements, and then adjust myself to compete again.*” Respondent 02 recalled: “*I would put the gun down, take two deep breaths, and then raise the gun again.*” Athletes will apply the strategy of “attentional shifting” (for example, deep breathing and pinching themselves) during the competition and then the strategy of “attentional focusing” on the sport task.

### Level 2 coding: identifying the core category

Due to the particularity of the ever-changing situation of competition, the existence of various MW sources will lead to athletes’ competition anxiety, making athletes’ MW more common. The MW may include worrying about losing the race, keeping track of scores, prizes, rankings, relatives, coaches, and eating and drinking. MW can take many different contents. Athletes will use attention control to reduce its occurrence. However, not all MW is meta-conscious, and sometimes athletes are not aware that MW is occurring. Human attentional resources are limited, and athletes require attentional resources for sport tasks and MW. Therefore, the attentional resources used during MW will inevitably impact the execution of the sport task, and this impact may even lead to abnormal performance. However, athletes believe that MW can sometimes have a positive effect on them. As 17 explained: “*Thinking about other things relaxes the slight tension and makes it less intense.*” But its negative effects are also exist. As 25 illustrated: “*MW in a game is more constraining to the sport tasks.*” Respondent 50 also described: “*Originally you could hit 9 or 10 rings, but if MW occurs, you may only hit 7, 8, or 6 rings.*”

We then conceptualized these stories to form the core category of this study. The story revolves around the phenomenon of MW in competition, and in any case, the devastating results of MW in a competitive situation are too much for the athlete to bear. We summarize this as “costs over benefits.” This category is not among the aforementioned categories but rather transcends them and consists of them. Therefore, this core category is structural, like all other categories become part of an architecture. Given the properties and dimensions of the core category are made up of other categories, they will not be repeated here, and the process by which they are influenced will be explained in detail in the theory formation section of this article.

### Level 3 coding: applying systems thinking to associate categories

**FIGURE 2:**
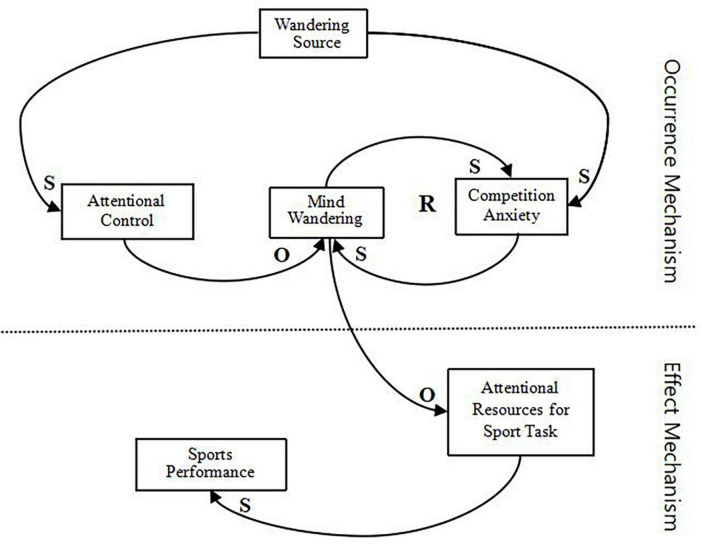
Mind wandering theory in the competition. S, S-type, the growth of one variable leads to the growth of another variable; O, O-type, the growth of one variable leads to the decline of another variable; R, an even number of O-types is an R-loop.

We innovate the theory formation method by combining the systemic thinking approach to relate the categories, resulting in “MW in sporting performance (MWSP)” (Figure 2). MWSP comprises two parts: occurrence mechanism and influence mechanism. The core idea is that MW in competition is influenced by the dynamics of MW source, competition anxiety, content of MW, attentional resources, and attentional control.

#### Occurrence mechanism

Athletes show higher attentional control when a competition has more MW sources and lower attentional control when a competition has fewer MW sources. The direction of change between the two variables was the same (S-type). When the athlete shows higher attentional control in the competition, the occurrence of MW decreases, and lower attentional control increases the occurrence of MW, with the direction of change between the two variables being opposite (O-type).

##### Mind wandering theory in competition

When a competition has more MW sources, the level of competition anxiety increases. When fewer MW sources are present, the level of competition anxiety decreases. The direction of change between the two variables is the same (S-type). Then, competition anxiety and MW will interact with each other, and the increase or decrease of competition anxiety level will lead to the increase or decrease of MW, and the direction of change between the two variables is the same (S-type). Here, a loop is formed, consisting of two S-type connections, which is an enhancement process, thus forming an “R-loop.”

#### Effect mechanism

The occurrence of MW occupies attentional resources, thus reducing attentional resources for sport tasks, and the two variables do not change in the same direction (O-type). The intensity of the change in the O-type is determined by content of MW. The content of MW has different attributes, which lead to a difference in the attention resources occupied by athletes and the attention resources for sport tasks.

The attentional resources for sport tasks in turn affect performance. When the attentional resources for sport tasks are reduced, the resources are not able to sustain the task during the competition, and performance will decrease. The two variables change in the same direction (S-type).

## Discussion

This study formed MWSP by grounded theory and systems thinking based on interview data from 51 athletes. MWSP was proposed for the first time in the context of competition, providing a theoretical basis for future research on MW in sports.

### Occurrence of MW in competition

The results of this study suggest that the occurrence of MW in competition is decided by MW sources, competition anxiety, and attentional control.

The MW sources include interference from others, time constraints, performance fluctuations, and environmental acceptance, which more directly reflect the situational specificity of MW during competition. Future intervention studies on MW could first attempt to simulate training on the above sources of MW. The MW sources in competition cannot be avoided, let alone predicted. Therefore, coaches need to conduct targeted simulation training based on these MW sources in daily training to improve athletes’ adaptability to MW sources.

Competition anxiety and MW form a positive feedback mechanism, which is reinforcing feedback (R-loop). First, anxiety triggers a higher frequency of MW in athletes. At present, no direct research evidence exists regarding the relationship between anxiety and MW in sports situations. However, some studies have shown that negative emotions lead the mind to wander (Smallwood et al., 2009). Anxiety, as one type of negative emotion, has been shown to have a significant correlation with MW (Seli et al., 2019). Does a high frequency of MW lead to increased levels of anxiety in athletes? The present study found that MW also can lead to competition anxiety. Some studies have confirmed that MW in daily life can lead to negative emotions (Killingsworth and Gilbert, 2010). But such a result has not emerged from any empirical study in the athlete population. Anxiety and MW are reciprocal influence relationships. Similarly, the relationship between anxiety and other internal thoughts with each other has been found in the field of athletics. For example, self-talk and MW are both internal thoughts of an individual. Research has shown that negative self-talk in sports predicted negative situational self-talk in competition and somatic and cognitive anxiety. In turn, cognitive anxiety positively predicted negative situational self-talk (Santos-Rosa et al., 2022). The second inspiration in sport practice is that the occurrence of MW can be reduced by reducing competition anxiety, ultimately minimizing its negative impact on sport performance, which is an issue worth exploring in the future.

Increased attentional control during competition will reduce the occurrence of MW. The “executive control failure hypothesis” and the “resource control hypothesis” emphasize the importance of attentional control. The executive control failure hypothesis suggests that MW is caused by a failure of executive control to maintain attention on the task (McVay and Kane, 2010). According to resource control theory, executive control weakens with the increase in the duration of performing sustained attention tasks (Thomson et al., 2015). The executive control failure hypothesis mainly emphasizes that attentional control is the cause of MW. The resource control hypothesis further elucidates the characteristics of attention control over time when performing alertness tasks. However, the MWSP, taking into account the special nature of the competition situation, does not believe that the ability to attentional control diminishes over the course of the game. This is because resource control theory explains a single sustained attention task, and games do not always require sustained attention. Notably, all of these theories believe that attentional control has an important role in the occurrence of MW. One theory discussing the relationship between competition anxiety, attentional control, and athletic performance proposed the Athletic Attentional Control Theory: Sport (ACTS). ACTS suggests that anxiety affects sport performance, and sport performance in turn affects anxiety (Eysenck and Wilson, 2016). ACTS also suggests that anxiety interferes with attentional control. Does anxiety interfere with attentional control followed by triggering MW? From this perspective, MWSP can be considered an extension of ACTS. Future research can be done with a third way to reduce the occurrence of MW in competition: to train attentional control.

However, we need the most fundamental solution of the above three ways to reduce the occurrence of MW. The occurrence mechanism of MWSP explains that competition anxiety and attentional control have different effects on MW: competition anxiety has a “fuel” effect on MW, such that anxiety triggers a higher frequency of MW in athletes. By contrast, attentional control reduces MW and acts as a “brake” on MW. Therefore, reducing competition anxiety and increasing attentional control can reduce MW during a competition. However, Figure 2 shows that the influence of competition anxiety and attentional control can be traced back to the MW source, which means that the root of MW occurs in the competition: the MW source. Maybe the first of these intervention methods, namely, simulation training for MW sources, is the fundamental solution, but future studies are needed to confirm it.

### Effect of MW in competition

Smallwood and Schooler (2015) have proposed that valuable topics for future research on MW include the impact of the characteristics of MW on the task. However, the prerequisite for exploring this topic is to clarify the contents in the mind when it wanders. The results of the present study showed that the contents of MW in athletes have 7 attributes and 15 dimensions. This reminds subsequent researchers of the importance of distinguishing the different contents of MW. If such distinction is neglected, erroneous conclusions may be drawn. For example, the type of intentional and unintentional MW has a different relationship with metacognition and self-awareness (Li et al., 2017; Vannucci and Chiorri, 2018), suggesting that subsequent researchers should consider them separately. The present study also showed that different contents of MW have different effects on athletes. However, athletes’ perceptions of the impact of a single attribute content were not consistent. For example, some athletes perceived a negative impact of irrelevant competition, but others perceived a positive impact. We argue that the correct judgment of the impact cannot be made by considering only a single dimensional content of MW nor by ignoring the characteristic (mainly contents and temporal context) of the task. Different content of MW and task features both occupy limited attentional resources. Correct judgment depends on the attentional resources reserved for the task at the time of MW.

The occurrence of MW takes up attentional resources, which is supported by the “decoupling hypothesis.” The decoupling hypothesis explains MW in terms of the allocation of attentional resources and suggests that MW is caused by the coupling of attention with internal processing while decoupling it from task-related information (Smallwood, 2010). The present study argues that the different contents of MW during competition lead to differences in the attentional resources they occupy, in turn affecting in different ways the attentional resources required for sports. For example, athletes with longer and deeper MW may consume more attentional resources, whereas those with relatively shorter and shallower MW may consume fewer attentional resources. This will ultimately affect the attentional resources allocated to a task and then affect sporting performance. In addition, the characteristics of the task performed in MW also need to be considered. For example, when MW occurs during a cognitively dominant task or at critical moments that determine athletic performance, ensuring the successful completion of the competition at this time may require more attentional resources for this kind task. At this time, there is a dynamic process of competing for attentional resources between MW and task. However, the exact amount of attentional resources taken up by different content of MW could not be confirmed in this study. This amount needs to be verified in future empirical studies (cognitive neuroscience approach may be the best solution), representing a research challenge that needs to be broken through. This study did find that the content of MW is a key factor affecting performance. In practice, if athletes cannot avoid MW on the field, they need to ensure that they avoid or reduce the use of attentional resources required for sport. Therefore, the method of managing MW content can change the attention resources occupied by MW, thereby reducing the negative impact and promoting the positive impact of MW on sports performance.

Additionally, in a single sporting event, the task performed by the athlete is variable, which will affect the occurrence of MW (as tennis doubles players 40 said: “they will be MW while waiting, and little or no MW will be allowed when the ball comes over”). If the more attentional resources are required for the execution of the task, the occurrence of MW at this time may result in limited attentional resources not being able to sustain the task, and then the negative effects of MW will be greater. Comparatively, skill-dominant sporting event may take up more attentional resources relative to physical-dominant. But, it is ultimately determined by the cognitive resources required for the varying tasks during the competition.

Theory of Challenge and Threat States in Athletes (TCTSA) also explores the effects of different states on athletic performance. The basic assumption of the TCTSA theory is that athletes have a dichotomous evaluation of upcoming competitions, either as a challenge or as a threat. The entire evaluation process is based on the athlete’s evaluation and comparison of demands and resources. Athletes are more likely to enter a challenge state when their perceived resources are greater than their demands, and more likely to enter a threat state when their perceived resources are insufficient to meet their demands (Jones et al., 2009). However, the TCTSA explores the challenge-threat state from a more macroscopic perspective (cognitive, emotional and physiological), which is essentially a motivational state. Meanwhile, the TCTSA addresses “resources” including self-efficacy ratings, perception of control, and goal orientation; while the MWSP explores the effects of a type of thought state on performance, where “resources” refers exclusively to attentional resources. In conclusion, helping athletes to achieve ideal performance and win in competition is one of the main tasks of sport psychologists. Theories are explored from different perspectives, but with the same goal of contributing to competition.

### Limitations and future directions

This study has some that need to be improved upon in the future. This study was able to obtain general results for athletes of multiple sporting events. These findings will undoubtedly be of value to athletes in many sports, but taking the whole into account will lead to a loss of specialization. We suggest that the hypotheses of this study be tested in future research for a single sport specialization. This will not only test the theory of this study but also provide assurance that the practice of this specialization will be correctly guided. In addition, the grounded theory that we have refined to meet the needs of our research is a risky endeavor that can create difficulties in understanding the results of the theory. The results of the study indicate that the relationships between the variables in the theory we present are directional and dynamic. This way of constructing and presenting the results of the theory is more informative and beneficial to the formation of clear research ideas for future empirical studies. Future research on grounded theory can also make methodological improvements based on research needs, contributing to qualitative methodology and yielding exciting results.

In conclusion, this study is based on the competition situation, rooted in original data, to form MWSP. The result has a theoretical dialogue with the executive control failure hypothesis, the resource control hypothesis, and the decoupling hypothesis. MWSP suggests that MW occupies attentional resources and emphasizes the role of attentional control. On this basis, we also found the important influence of MW sources, competition anxiety, and content of MW. In daily training, we can learn from the occurrence and influence mechanisms of MWSP. Coaches can apply the intervention method of simulating the MW sources to reduce the frequency of MW in competition. At the same time, the negative effects can be mitigated and positive effects can be promoted by managing the content of MW.

## Conclusion

To investigate the reasons why athletes’ MW affects performance, this study followed grounded theory and combined systems thinking to propose MWSP. The theory suggests that MW affecting sporting performance is influenced by the dynamics of MW source, competition anxiety, content of MW, attentional resources, and attentional control. MWSP is able to explain MW’s bidirectional effects and provides scientific guidance for the intervention of athletes’ MW. The hypothesis in this theory can be empirically tested in the future to examine its explanatory power.

## Data availability statement

The raw data supporting the conclusions of this article will be made available by the authors, without undue reservation.

## Ethics statement

The studies involving humans were approved by the Ethics Committee of Hebei Normal University (2023LLSC031). The studies were conducted in accordance with the local legislation and institutional requirements. The participants provided their written informed consent to participate in this study.

## Author contributions

JL: Conceptualization, Data curation, Funding acquisition, Methodology, Project administration, Supervision, Writing – original draft. YL: Data curation, Validation, Writing – original draft. SX: Data curation, Writing – review & editing. BT: Conceptualization, Methodology, Project administration, Validation, Writing – review & editing.

## Footnotes

1
https://baijiahao.baidu.com/s?id=1724296430892529343&wfr=spider&for=pc

2
https://www.chinanews.com/olympic/news/2008/08-17/1350776.shtml
